# Mental health and academic performance in a cohort of first year primary school children in Chile

**DOI:** 10.1192/j.eurpsy.2022.1067

**Published:** 2022-09-01

**Authors:** M. Rivas, G. Gómez, V. Giaconi, M.S. Burrone

**Affiliations:** 1 Universidad de O’Higgins, Institute Of Educational Sciences, Rancagua, Chile; 2 Universidad de O´Higgins, Institute Of Health Sciences, Rancagua, Chile

**Keywords:** Child, mental health, Academic attainment, Chile

## Abstract

**Introduction:**

Psychiatric disorders are common in children, and academic attainment is lower in children with psychiatric disorders. There are few data about the occurrence of mental health problems and the academic attainment among children in Chile.

**Objectives:**

To determine the occurrence of mental health problems and its association with academic attainment in first-year students of elementary schools in Chile

**Methods:**

The study was conducted in 39 urban and rural public elementary schools in Chile in 2019. The academic performance was measured using the Woodcock Muñoz IV Battery and the Corsi Bock-Tapping test. Mental health was assessed using the self-report Dominique Interactive and Strengths in children and Difficulties Questionnaire (SDQ) instruments in their parents and teachers. A triangulation of information was conducted between different informants.

**Results:**

Overall 610 children were included in the analysis (mean age 7.10 years (SD=0.58), 51% women, 36% from rural area). A higher score in mathematics and reading was negatively correlated to a higher score in emotional symptoms, hyperactivity and peer relationship difficulties, separately, based on both teacher- and parent-reported SDQ. The correlation coefficient between reading scores with Dominique Interactive and Strengths externalizing symptoms was -0.22 (p<0,05). A higher maternal education level was associated with higher education attainment in their children (p <0,05). There was no association between rurality and children’s mental health symptoms.

**Conclusions:**

The current results can inform local stakeholders in Chile about the importance of mental health at a very early age.

**Disclosure:**

No significant relationships.

